# Cardiovascular effects of methylphenidate, risperidone, and their combination in children and adolescents with attention-deficit/hyperactivity disorder: a prospective 3-month observational study

**DOI:** 10.1186/s12887-026-07479-x

**Published:** 2026-08-06

**Authors:** Sonia Oveisi, Elahe Jafari, Mohamad Mahdi Bahari, Mina Farshidgohar

**Affiliations:** 1 Clinical Research Development Unit of Advanced Medicine, Qazvin University of Medical Science, Qazvin, Iran; 2 Department of Epidemiology, School of Public Health, University of Medical Sciences, Tehran, Iran; 3 Non-communicable Diseases Research Center, Research Institute for Prevention of Non-communicable Diseases, Qazvin University of Medical Sciences, Qazvin, Iran; 4 Students’ Research Committee, Qazvin University of Medical Sciences, Qazvin, Iran

**Keywords:** Attention Deficit Disorder with Hyperactivity, Electrocardiography, Hemodynamic Monitoring, Methylphenidate, Risperidone

## Abstract

**Background:**

We aimed to evaluate changes in hemodynamic parameters and electrocardiographic (ECG) indices related to ventricular arrhythmia risk after three months of treatment with methylphenidate (MPH), risperidone (RIS), or their combination in children and adolescents with ADHD.

**Methods:**

A total of 150 children and adolescents with DSM-5-diagnosed ADHD were prospectively enrolled at Qods Hospital. Participants received methylphenidate (*n* = 50), risperidone (*n* = 50), or combination therapy (*n* = 50). Cardiovascular outcomes were assessed using age- and weight-adjusted two-way repeated-measures ANCOVA to evaluate changes over time and differences among treatment groups.

**Results:**

Significant group × time interactions were observed for systolic blood pressure, diastolic blood pressure, heart rate, and QTc interval after adjustment for baseline age and weight. After three months, systolic and diastolic blood pressure increased in the methylphenidate and combination therapy groups, while heart rate showed an overall increase, with the greatest rise observed in the methylphenidate group. QTc interval increased over time across all treatment groups, with the largest prolongation observed in the combination therapy group (377.9 ± 25.4 to 424.5 ± 24.9 ms). However, mean QTc values remained below commonly accepted thresholds for clinically significant QT prolongation, and no significant longitudinal changes were observed in QRS duration or PR intervals.

**Conclusion:**

Treatment with methylphenidate, risperidone, and combination therapy was associated with modest changes in heart rate, blood pressure, and QTc interval over three months. These findings highlight the importance of baseline and follow-up cardiovascular monitoring in children and adolescents receiving ADHD medications.

## Introduction

 Attention-deficit/hyperactivity disorder (ADHD) is one of the most common neurodevelopmental disorders in childhood. A worldwide systematic review and meta-regression analysis estimated the pooled prevalence of ADHD among children and adolescents at approximately 5.3%, highlighting its substantial public health burden [1]. ADHD is characterized by developmentally inappropriate and impairing levels of inattention and/or hyperactivity-impulsivity that interfere with daily functioning and social, academic, and occupational performance [2].

Diagnostic classifications for ADHD have evolved over time, with differences between major international systems. In the 11th revision of the International Classification of Diseases (ICD-11), the World Health Organization adopted the term “attention deficit hyperactivity disorder” (ADHD), replacing the previous terminology of “hyperkinetic disorder” used in earlier ICD classifications [3]. Although ADHD diagnostic criteria in ICD-11 and DSM-5-TR are largely comparable, some differences exist regarding symptom thresholds, clinical presentations, and diagnostic requirements [4]. These diagnostic frameworks describe overlapping clinical constructs, while ADHD remains the preferred terminology in contemporary clinical practice [4].

The use of pharmacological treatments for ADHD has increased substantially worldwide over recent decades, with notable rises in medication prescribing among both children and adults [5]. Although randomized clinical trials and observational studies have demonstrated the effectiveness of ADHD medications in reducing core symptoms and improving functional outcomes, concerns regarding their long-term safety, particularly potential cardiovascular effects, remain an important clinical consideration [6–8].

Pharmacological treatment of ADHD includes both stimulant and non-stimulant medications. According to current clinical guidelines, stimulant medications, primarily methylphenidate- and amphetamine-based preparations, are considered first-line pharmacological options for many children and adolescents with ADHD. Non-stimulant medications, including atomoxetine, guanfacine, and clonidine, represent alternative treatment options, particularly for patients with inadequate response to stimulants or those experiencing adverse effects [8, 9].

Methylphenidate (MPH), a central nervous system stimulant, is one of the most commonly used medications for ADHD, whereas risperidone (RIS), an atypical antipsychotic, is frequently prescribed for children and adolescents with ADHD-associated behavioral disorders. These medications may be administered as monotherapy or combination therapy when clinically indicated. Although effective, they may be associated with neurological, metabolic, and cardiovascular adverse effects [7–9]. Cardiovascular effects are of particular clinical importance in pediatric populations because stimulants and antipsychotics may influence heart rate, blood pressure, and cardiac electrophysiological parameters. Previous studies have reported alterations in ECG parameters, including QT and corrected QT (QTc) interval prolongation, which may increase the risk of ventricular arrhythmias in susceptible individuals [10–14].

Therefore, careful assessment of ventricular repolarization parameters is essential, particularly in children receiving combined therapy. The present study was designed to investigate changes in ECG parameters and hemodynamic variables among patients with ADHD receiving MPH, RIS, or combination therapy.

## Methods

This prospective observational cohort study was conducted in Qazvin, Iran, from October 2024 to January 2025 and included 150 children and adolescents aged 4–15 years diagnosed with ADHD. ADHD was diagnosed by a board-certified child and adolescent psychiatrist in accordance with the Diagnostic and Statistical Manual of Mental Disorders, Fifth Edition (DSM-5) [15]. The diagnostic process was based on a comprehensive clinical evaluation, including interviews with the child and parents, review of the child’s developmental and medical history, and assessment of behavioral symptoms across different settings whenever possible. As this was an observational study conducted in routine clinical practice, standardized rating scales such as the Conners Rating Scale were not routinely used, and formal assessments of ADHD severity were not performed. In addition, ADHD presentations (predominantly inattentive, predominantly hyperactive-impulsive, or combined) were not consistently documented and were therefore not included in the analysis.

Only children and adolescents with a newly diagnosed with ADHD were included in the study. None of the participants had received previous pharmacological treatment for ADHD, and patients with comorbid psychiatric disorders were excluded to minimize potential confounding effects on the study outcomes.

Participants were classified into three treatment groups (MPH (*n* = 50), RIS (*n* = 50) and MPH + RIS (*n* = 50)).

Patients in the methylphenidate group received immediate-release methylphenidate at an initial dose of 0.5 mg/kg/day. Patients in the risperidone group started treatment with 0.5 mg/day, which was the standard initial dose routinely used in our clinic during the study period. Participants in the combination group received both medications using the same initial dosing strategy. The initial doses were maintained throughout the three-month follow-up period. As the study was designed to evaluate the short-term cardiovascular effects of treatment, no dose adjustments were made during the study. Any clinically indicated dose modifications were considered only after completion of the three-month follow-up, in accordance with routine clinical practice.

Although immediate-release methylphenidate is generally recommended for children aged 6 years and older, a small number of children younger than 6 years received this medication based on the clinical judgment of a board-certified child and adolescent psychiatrist. Treatment decisions were individualized following a comprehensive clinical assessment, taking into account symptom severity, functional impairment, and the expected benefits and potential risks of treatment.

Sample size calculation: The sample size was calculated using G*Power software (version 3.1). Assuming a medium effect size (Cohen’s f = 0.25), a significance level of 0.05, and a statistical power of 80% for comparing three treatment groups, the required total sample size was estimated to be approximately 150 participants. Therefore, 150 patients were enrolled, with approximately 50 participants included in each treatment group.

### Participants

Children and adolescents diagnosed with ADHD were eligible for inclusion if they met the following criteria: (i) Iranian nationality, confirmed by official identification documents; (ii) age between 4 and 15 years; and (iii) clinical indication for treatment with MPH, RIS, or their combination, as determined by a board-certified child and adolescent psychiatrist according to standard clinical guidelines and recommended dosing regimens.

Exclusion criteria included a history of congenital heart disease, patients with comorbid psychiatric disorders, baseline QTc prolongation or known cardiac arrhythmias, significant systemic illnesses, or concurrent use of medications with known cardiotoxic effects. These conditions were assessed through detailed medical history, clinical examination, review of medical records, and baseline ECG evaluation.

### Follow-up procedures

Follow-up assessments for both monotherapy and combination therapy were conducted according to standard clinical practice guidelines for the management of ADHD in children and adolescents, including regular psychiatric evaluations and systematic monitoring of treatment response, adverse effects, and medication adherence [16]. Patients were routinely evaluated at baseline and at scheduled follow-up visits every 4 weeks during the study period.

No participants were lost to follow-up during the study period, as all enrolled patients completed the scheduled clinical visits. In cases of missed appointments, patients were actively contacted by telephone, and follow-up visits were rescheduled within one week to ensure complete data collection.

### Data collection and measurements

We performed a descriptive assessment of the demographic characteristics and cardiovascular parameters of the children and adolescents included in our study. Baseline information was collected for all participants, including age, sex, and body mass index (BMI). Cardiovascular monitoring consisted of ECG measurements, blood pressure recordings, and heart rate evaluation.

A standard 12-lead ECG was performed for all participants at baseline and repeated three months after treatment initiation. All ECG recordings were obtained and interpreted by a board-certified pediatric cardiologist. QRS duration, PR interval, and corrected QT (QTc) interval were measured using standardized procedures, ensuring accuracy and consistency across all participants. The corrected QT interval (QTc) was calculated using Bazett’s formula (QTc = QT/√RR), with both QT and RR intervals measured in seconds.

Systolic and diastolic blood pressure (BP) were measured at baseline and at the three-month follow-up using an automated pediatric sphygmomanometer. Measurements were performed according to standard pediatric protocols: participants were seated quietly for at least five minutes before measurement, and the appropriate cuff size was used. Three consecutive readings were obtained at each assessment point, and the average value was recorded. Heart rate was monitored simultaneously during each BP measurement. All measurements were conducted by trained clinical staff under the supervision of a pediatric cardiologist to ensure consistency and accuracy. Body mass index (BMI) was calculated as weight in kilograms divided by height in meters squared (kg/m²) at baseline and at the three-month follow-up. Weight was measured using a calibrated digital pediatric scale, and height was measured with a stadiometer, both recorded to the nearest 0.1 kg and 0.1 cm, respectively. Measurements were performed in light clothing and without shoes, and each measurement was taken twice with the average value used for BMI calculation.

### Statistical analysis

Data were entered, managed, and analyzed using SPSS version 26 (IBM Corp., Armonk, NY, USA). Continuous variables are presented as mean ± standard deviation (SD), whereas categorical variables are presented as frequencies and percentages. Baseline demographic, anthropometric, and cardiovascular characteristics were compared among the three treatment groups using one-way analysis of variance (ANOVA) for continuous variables and the chi-square (χ²) test for categorical variables. Between-group comparisons at the 3-month follow-up were performed using one-way ANOVA. Longitudinal changes in electrocardiographic and hemodynamic parameters were assessed using age- and weight-adjusted two-way repeated-measures analysis of covariance (ANCOVA), with treatment group as the between-subject factor and time (baseline and 3-month follow-up) as the within-subject factor. The group × time interaction term was used to determine whether changes over time differed significantly among the treatment groups after adjustment for baseline age and weight. Effect sizes were reported as partial eta squared (ηp²). A two-sided P value < 0.05 was considered statistically significant.

## Results

A total of 150 children and adolescents aged 4–15 years were included in the study, comprising 94 boys (62.7%) and 56 girls (37.3%). The mean BMI of the study population was 22.30 ± 1.90 kg/m². Baseline demographic, Baseline demographic, anthropometric, and clinical characteristics according to treatment group are presented in Table 1.Significant differences among the three treatment groups were observed in age (*P* < 0.001), weight (*P* = 0.001), and PR interval (*P* = 0.029). In contrast, sex distribution, BMI, systolic and diastolic blood pressure, heart rate, QRS duration, and QTc interval showed no significant differences among the groups at baseline (all *P* > 0.05).

**Table 1:** Baseline demographic, anthropometric, and cardiovascular characteristics by treatment group

Parameter	RIS	MPH	Combination	*P* value†
Age (years)	8.62 (2.73)	10.84 (2.46)	9.74 (3.11)	< 0.001
Male, n (%)	35 (37.2)	28 (29.8)	31 (33.0)	0.121‡
Weight (kg)	35.32 (13.86)	42.37 (12.19)	39.20 (14.18)	0.001
BMI (kg/m²)	22.18 (1.97)	22.28 (1.83)	22.45 (1.91)	0.585

† P values represent between-group comparisons at baseline using one-way ANOVA for continuous variables

‡ P value for sex was obtained using the χ² test

### Electrocardiographic findings

Changes in electrocardiographic parameters from baseline to the 3-month follow-up are presented in Table 2. After adjustment for baseline age and weight using repeated-measures ANCOVA, a significant group × time interaction was observed for QTc interval (*P* = 0.002, partial η² = 0.041). QTc intervals increased in all three treatment groups during follow-up. In the risperidone group, QTc increased from 370.76 ± 20.95 ms at baseline to 413.00 ± 27.58 ms after three months. In the MPH group, QTc increased from 373.38 ± 24.13 ms to 396.54 ± 27.56 ms, whereas the combination therapy group showed the largest increase, from 377.94 ± 25.38 ms to 424.54 ± 24.89 ms.

**Table 2:** Effects of Methylphenidate, Risperidone, and combination therapy on electrocardiographic findings in baseline and after 3 months follow up

Parameter	Drug Group	Mean ± SD (ms) Baseline	Mean ± SD (ms) After three months	Group × Time interaction *P*-value	Partial Eta Squared
QRS	RIS	76.80 ± 11.33	76.60 ± 14.65	0.580	0.004
	MPH	72.60 ± 16.26	74.80 ± 16.07		
	Combination	73.60 ± 13.37	77.60 ± 12.87		
PR interval	RIS	112.00 ± 14.14	108.20 ± 13.66	0.210	0.010
	MPH	106.20 ± 16.65	109.10 ± 15.51		
	Combination	103.40 ± 17.8	106.80 ± 18.56		
QTc	RIS	370.76 ± 20.95	413.00 ± 27.58	0.002	0.041
	MPH	373.38 ± 24.13	396.54 ± 27.56		
	Combination	377.94 ± 25.38	424.54 ± 24.89		

*RIS* Risperidone, *MPH* Methylphenidate

*P*-values and partial eta squared values were derived from repeated-measures ANCOVA adjusted for baseline age and weight. Values are presented as mean ± standard deviation

No significant group × time interaction was observed for QRS duration (*P* = 0.577, partial η² = 0.004) or PR interval (*P* = 0.214, partial η² = 0.010). QRS duration remained relatively stable across all treatment groups. PR intervals showed minor changes over time, with a slight reduction in the risperidone group and small increases in the methylphenidate and combination therapy groups.

## Hemodynamic Findings

Changes in hemodynamic parameters are presented in Table 3. After adjustment for baseline age and weight, significant group × time interactions were observed for systolic blood pressure (*P* < 0.001, partial η² = 0.103) and diastolic blood pressure (*P* < 0.001, partial η² = 0.119).

**Table 3:** Effects of Methylphenidate, Risperidone, and combination therapy on blood pressure, and heart rate findings in baseline and after 3 months follow up

Parameter	Drug Group	Mean ± SD (ms) Baseline	Mean ± SD (ms) After three months	Group × Time interaction *P*-value	Partial Eta Squared
Systolic BP(mmHg)	RIS	103.40 ± 10.62	101.80 ± 9.99	< 0.001	0.103
	MPH	99.90 ± 9.06	110.20 ± 8.33		
	Combination	103.50 ± 10.11	111.00 ± 10.25		
Diastolic BP (mmHg)	RIS	73.40 ± 7.72	72.03 ± 8.03	< 0.001	0.119
	MPH	71.60 ± 6.88	80.20 ± 3.64		
	Combination	73.40 ± 7.66	80.60 ± 4.12		
HR	RIS	75.10 ± 7.18	82.66 ± 7.89	0.047	0.026
	MPH	78.08 ± 7.87	91.04 ± 8.39		
	Combination	75.34 ± 6.69	85.52 ± 7.94		

*RIS* Risperidone, *MPH* Methylphenidate

*P*-values and partial eta squared values were derived from repeated-measures ANCOVA adjusted for baseline age and weight. Values are presented as mean ± standard deviation

Systolic blood pressure decreased slightly in the risperidone group, from 103.40 ± 10.62 mmHg at baseline to 101.80 ± 9.99 mmHg after three months. In contrast, significant increases were observed in the methylphenidate group (99.90 ± 9.06 to 110.20 ± 8.33 mmHg) and the combination therapy group (103.50 ± 10.11 to 111.00 ± 10.25 mmHg).

A similar pattern was observed for diastolic blood pressure. Values remained relatively unchanged in the risperidone group (73.40 ± 7.72 to 72.03 ± 8.03 mmHg), whereas increases were observed in the methylphenidate (71.60 ± 6.88 to 80.20 ± 3.64 mmHg) and combination therapy groups (73.40 ± 7.66 to 80.60 ± 4.12 mmHg).

Heart rate increased across all treatment groups during follow-up. The greatest increase was observed in the methylphenidate group (78.08 ± 7.87 to 91.04 ± 8.39 beats/min), followed by the combination therapy group (75.34 ± 6.69 to 85.52 ± 7.94 beats/min) and the risperidone group (75.10 ± 7.18 to 82.66 ± 7.89 beats/min). The group × time interaction was statistically significant for heart rate, although the effect size was small (*P* = 0.047, partial η² = 0.026).

## Discussion

The present study evaluated short-term cardiovascular changes associated with methylphenidate, risperidone, and their combination in children and adolescents with ADHD. The main findings indicate differential effects of these treatment regimens on electrocardiographic and hemodynamic parameters. QTc interval increased significantly during the three-month follow-up across all treatment groups, with the greatest increase observed in the combination therapy group. In contrast, PR interval and QRS duration remained unchanged, suggesting no clinically meaningful effects on atrioventricular or intraventricular conduction. Regarding hemodynamic parameters, methylphenidate-containing regimens were associated with increases in systolic and diastolic blood pressure and heart rate, whereas risperidone monotherapy showed minimal changes.

### Effect on ECG parameters

In the present study, a significant increase in QTc interval was observed during the three-month follow-up in all treatment groups, with the largest numerical increase observed in the combination therapy group. Although the group × time interaction for QTc was statistically significant, no immediate clinically significant consequences were observed during the study period. Only two participants receiving combination therapy reached QTc values of 460 ms, while no participant developed QTc prolongation ≥ 500 ms, clinically significant arrhythmias, or required treatment discontinuation or cardiac intervention.

QTc prolongation represents delayed ventricular repolarization and may increase the risk of malignant ventricular arrhythmias, particularly in the presence of additional risk factors, including electrolyte disturbances, congenital long QT syndrome, structural heart disease, or concomitant use of other QT-prolonging medications [11–13]. Therefore, periodic ECG monitoring may be considered in children receiving combined methylphenidate and risperidone therapy, particularly during prolonged treatment or among patients with additional cardiovascular risk factors.

In contrast, no significant group × time interaction was observed for PR interval or QRS duration, indicating that short-term exposure to methylphenidate, risperidone, or their combination did not result in clinically meaningful alterations in cardiac conduction. These findings are consistent with previous evidence from Karpuz et al., who evaluated methylphenidate, risperidone, and combination therapy in children with ADHD and reported no significant adverse effects on conventional ECG conduction parameters [10].

Previous studies investigating methylphenidate-related cardiovascular effects have generally reported limited effects on standard ECG indices. Şimşek et al. found that three months of methylphenidate treatment in children with ADHD was not associated with significant changes in QTc interval or conventional ECG parameters, although subtle changes in ventricular repolarization indices, including Tp–Te and Tp–Te/QTc ratios, were observed [17]. Similarly, Lamberti et al. reported that a single therapeutic dose of immediate-release methylphenidate did not significantly alter QTc interval or QT dispersion, despite minor changes in repolarization-related markers [18]. Furthermore, Isart et al. suggested that alternative ventricular repolarization indices, such as JTc interval, may provide additional information beyond QTc assessment in children with ADHD [19].

Collectively, available evidence suggests that ADHD medications may produce measurable changes in ventricular repolarization and hemodynamic parameters; however, clinically significant arrhythmias and serious cardiovascular outcomes remain uncommon when treatment is appropriately prescribed and cardiovascular risk factors are monitored [9, 20, 21]. Nevertheless, the significant QTc increase observed in the present study, particularly among patients receiving combination therapy, supports the importance of continued ECG surveillance when multiple psychotropic medications are used concurrently.

### Effect on hemodynamic parameters

The present study demonstrated significant changes in blood pressure and heart rate following three months of treatment with methylphenidate, risperidone, or their combination. Methylphenidate monotherapy and combination therapy were associated with significant increases in both systolic and diastolic blood pressure, whereas risperidone monotherapy showed minimal changes over time. In the methylphenidate and combination groups, systolic blood pressure increased by approximately 8–10 mmHg, while diastolic blood pressure increased by approximately 7–9 mmHg. Heart rate also increased across all treatment groups, with the greatest numerical increase observed in the methylphenidate group; however, the effect size was small (partial η² = 0.026).

These findings are consistent with previous evidence indicating that stimulant medications produce modest but measurable increases in cardiovascular parameters among children and adolescents with ADHD. A systematic review and meta-analysis of randomized controlled trials demonstrated that stimulant medications, including methylphenidate, were associated with small but statistically significant increases in systolic blood pressure, diastolic blood pressure, and heart rate, although the magnitude of these changes was generally limited [20]. Similarly, long-term follow-up data from the Multimodal Treatment Study of ADHD showed that stimulant exposure was associated with relatively small changes in blood pressure and heart rate over time, without evidence of clinically meaningful sustained cardiovascular deterioration in most patients [22].

Recent prospective studies further support these findings. Omidi et al. reported that three months of methylphenidate treatment in children with ADHD resulted in significant increases in blood pressure and heart rate, while cardiovascular changes remained within clinically acceptable ranges [14]. In addition, Jaicks and Yıldırım demonstrated that even acute administration of methylphenidate could increase systolic blood pressure, diastolic blood pressure, and heart rate shortly after treatment initiation, suggesting that these hemodynamic effects may occur early during exposure [21].

Although the absolute increases observed in our study were relatively modest, their clinical relevance should not be overlooked. Persistent elevations in blood pressure or heart rate may be clinically important in susceptible individuals, particularly those with pre-existing cardiovascular risk factors, obesity, or concomitant use of multiple psychotropic medications. Large observational studies and meta-analyses have generally shown that ADHD medications are not associated with increased risks of myocardial infarction, stroke, or overall mortality; however, a modest increase in the risk of arrhythmia cannot be completely excluded, emphasizing the need for appropriate cardiovascular assessment and monitoring during treatment [23, 24].

Overall, our findings support the view that methylphenidate-containing regimens, including combination therapy with risperidone, have an acceptable short-term cardiovascular safety profile when appropriately prescribed. Nevertheless, the observed increases in QTc interval, blood pressure, and heart rate highlight the importance of regular cardiovascular monitoring, especially among patients receiving long-term or combined pharmacological treatment.

### Strengths and limitations

A key strength of the present prospective study is the direct comparison of multiple ADHD treatment regimens, including RIS, MPH, and their combination, which allowed a comprehensive evaluation of their relative cardiac effects. The prospective design with standardized follow-up assessments, including repeated electrocardiographic and clinical measurements, enhances the reliability and internal validity of the findings. Additionally, careful monitoring of heart rate, blood pressure, and ECG parameters at baseline and during follow-up supports robust detection of treatment-related cardiac changes.

Several limitations should be considered when interpreting our findings. First, the follow-up period was limited to three months, which precludes evaluation of long-term cardiovascular outcomes. However, this duration was selected to assess early and subacute cardiovascular changes that may occur during the initial months of treatment with MPH, RIS, or their combination in pediatric populations.

Second, although medication doses were predefined according to routine clinical practice and maintained throughout the three-month follow-up period, we were unable to evaluate potential dose–response relationships or the impact of cumulative medication exposure on cardiovascular outcomes. In addition, detailed information regarding ADHD subtype, symptom severity, and treatment adherence was not available for all participants, limiting our ability to fully assess whether these factors influenced the observed cardiovascular changes.

Third, because treatment allocation was based on routine clinical practice rather than randomization, the study is susceptible to confounding by indication. In particular, patients receiving combination therapy may have represented a clinically more complex subgroup with greater symptom burden, behavioral difficulties, or inadequate response to monotherapy. Therefore, the greater changes observed in cardiovascular parameters, including QTc interval, may partly reflect underlying differences in clinical characteristics rather than the independent effect of combined pharmacological treatment. Although longitudinal analyses and adjustment for available baseline characteristics strengthen the findings, residual confounding due to unmeasured factors cannot be completely excluded. Thus, the results should be interpreted as associations rather than evidence of causality.

Fourth, the absence of a healthy control group or an untreated ADHD comparison group limits the ability to distinguish medication-related cardiovascular changes from normal developmental changes or temporal variations unrelated to treatment. Future controlled studies with appropriate comparison groups are needed to clarify the specific contribution of pharmacological exposure to cardiovascular alterations.

Fifth, the study included children and adolescents across a relatively wide age range (4–15 years). Because the sample size was insufficient for adequately powered age-stratified analyses, potential differences in cardiovascular responses across developmental stages could not be evaluated. Larger multicenter pediatric studies are needed to investigate age-specific effects of these medications.

Sixth, this was a single-center study conducted in a referral pediatric hospital, which may limit the generalizability of the findings to other populations and healthcare settings.

Seventh, although standard ECG parameters, including QTc interval, PR interval, and QRS duration, were assessed, other potentially informative markers of ventricular repolarization, such as T-wave morphology, Tp–Te interval, QT dispersion, and JTc interval, were not evaluated. Furthermore, QTc was calculated using Bazett’s formula, which may overestimate QTc values in individuals with higher heart rates. Future studies using alternative correction methods, such as Fridericia’s formula, may provide more accurate assessment of ventricular repolarization.

Eighth, although ECG measurements were reviewed by a pediatric cardiologist, the absence of blinding to treatment status may have introduced potential observer bias.

Finally, because height measurements were unavailable for all participants, age-, sex-, and height-specific blood pressure percentiles could not be calculated. Therefore, we were unable to determine the proportion of participants with blood pressure values exceeding the 95th percentile according to pediatric reference standards.

## Conclusion

Combination therapy was associated with greater QTc prolongation and blood pressure elevation compared with monotherapy. Although changes in heart rate were modest and cardiac conduction parameters remained stable, these findings highlight the importance of individualized cardiovascular assessment and appropriate monitoring, particularly in patients receiving combined psychotropic therapy or with additional cardiovascular risk factors.

## Data Availability

The data that support the findings of this study are available from the corresponding author upon formal request.
